# A Novel and Validated Protocol for Performing MIC Tests to Determine the Susceptibility of *Piscirickettsia salmonis* Isolates to Florfenicol and Oxytetracycline

**DOI:** 10.3389/fmicb.2017.01255

**Published:** 2017-07-06

**Authors:** Sergio Contreras-Lynch, Peter Smith, Paola Olmos, María E. Loy, William Finnegan, Claudio D. Miranda

**Affiliations:** ^1^Departamento de Salud Hidrobiológica, Instituto de Fomento PesqueroPuerto Montt, Chile; ^2^Department of Microbiology, School of Natural Sciences, National University of IrelandGalway, Ireland; ^3^College of Engineering and Informatics, National University of IrelandGalway, Ireland; ^4^Laboratorio de Patobiología Acuática, Departamento de Acuicultura, Universidad Católica del NorteCoquimbo, Chile

**Keywords:** *Piscirickettsia salmonis*, MIC protocol, epidemiological cut-off value, antimicrobial susceptibility, fish pathogen, salmon farming

## Abstract

This paper presents a validated protocol, using a novel, specifically formulated medium, to perform broth microdilution antimicrobial susceptibility assays of the salmonid bacterial pathogen *Piscirickettsia salmonis*. The minimum inhibitory concentrations (MIC) for florfenicol and oxytetracycline against 58 *P. salmonis* isolates recovered from various outbreaks occurred in Chilean salmonid farms were determined using this protocol. Normalized resistance interpretation (NRI) analysis was applied to these data to calculate appropriate protocol-specific epidemiological cut-off values. These cut-off values allow the isolates to be categorized as either fully susceptible wild type (WT) members of this species, or as manifesting reduced susceptibility non-wild type (NWT). The distribution of MIC values of florfenicol was bimodal and the distribution of the normalized values for the putative WT observation had a standard deviation of 0.896 log_2_ μg mL^-1^. This analysis calculated a cut-off value of ≤0.25 μg mL^-1^ and categorized 33 (56%) of the isolates as manifesting reduced susceptibility to florfenicol. For the oxytetracycline MIC data the NRI analysis also treated the distribution as bimodal. The distribution of the normalized values for the putative WT observation had a standard deviation of 0.951 log_2_ μg mL^-1^. This analysis gave a cut-off value of ≤0.5 μg mL^-1^ and categorized five isolates (9%) as manifesting reduced susceptibility to oxytetracycline. The susceptibility testing protocol developed in this study was capable of generating MIC data from all the isolates tested. On the basis of the precision of the data it generated, and the degree of separation of values for WT and NWT it achieved, it is argued that this protocol has the performance characteristics necessary for it to be considered as a standard protocol.

## Introduction

Piscirickettsiosis, the disease caused by the intracellular pathogenic bacterium *Piscirickettsia salmonis*, is currently the most important bacterial disease impacting seawater salmonid farming in Chile and is considered a serious threat to the sustainability of the salmon industry (Ibieta et al., 2011). Among the infectious causes of mortality in the Chilean salmon industry for Atlantic salmon in 2015, 78.9% were associated with Piscirickettsiosis (Sernapesca, 2016a).

Currently, there are no efficient commercial vaccines for the control of Piscirickettsiosis (Rozas and Enríquez, 2014) and consequently antibiotic therapy has been used extensively in sea farms to reduce losses due to this disease. In 2015, approximately 557.2 tons of antibiotics were used by the Chilean salmon industry and from these the 96% was administered in sea farms. Most of the antibacterials (94%) used in sea farms were administered to treat *P. salmonis* infections (Sernapesca, 2016b). Currently, florfenicol was the drug of choice to treat *P. salmonis* outbreaks during 2015, representing the 87% of antibiotics used, whereas oxytetracycline was the drug of second choice (12%) to treat this pathology (Sernapesca, 2016b). However, it has been observed that *P. salmonis* infected salmonids respond poorly or inconsistently to drug treatments. It has been suggested that these treatment failures may be related to the intracellular location of the bacterium and that treatments may not achieve a sufficient concentration of antibiotics to kill or inhibit the pathogen within the host cells (Mauel and Miller, 2002; Fryer and Hedrick, 2003). It is equally possible that reductions in the susceptibility of *P. salmonis* to the agents may be associated with some of the treatment failures reported.

Smith et al. (2008) have argued that if antibiotics are used prudently and in an economically rational manner it is essential that the selection of the antibiotic to be administered in each outbreak be informed by laboratory data on the susceptibility of the target bacterium. The Aquatic Animal Health Code of the World Animal Health Organisation^1^ has argued that the use of internationally harmonized and standardized protocols for susceptibility testing are essential if data produced in different laboratories are to be meaningfully compared. Such a standardized protocol is not currently available for the susceptibility testing of *P. salmonis*. The development, adoption, and implementation of such a standard protocol should be given a high priority.

As well as a standardized testing protocol the Aquatic Animal Health Code recommends the adoption of statistically based criteria (epidemiological cut-off values) for the interpretation of the meaning of the minimum inhibitory concentration (MIC) values generated by that protocol. Two methods are available for the setting of epidemiological cut-off values. These are the normalized resistance interpretation (NRI) method of Kronvall (2010), for which the current version is available on-line^2^ and the ECOFFinder method of Turnidge et al. (2006), available on-line^3^. These two analytical methods use different statistical approaches but the major difference is that NRI was specifically designed to deal with situations where the distribution of MIC values for isolates with a slightly reduced susceptibility were close to or overlapped with those fully susceptible isolates.

As the validity of any epidemiological cut-off value is proportional to the precision of the MIC data from which it was calculated (Smith et al., 2012), the ability to generate data of adequate precision is a very important aspect of a susceptibility test protocol, especially in cases such as this intracellular pathogenic species. *P. salmonis* is considered a fastidious bacterium because exhibit high nutrient requirements for its growth, and is unable to grow in classical standardized culture media recommended by the AST or CLSI (Clinical & Laboratory Standards Institute) guidelines such as Mueller–Hinton broth. Furthermore, the current CLSI guideline for determining the MIC values for bacteria isolated from aquatic animals (CLSI, 2014a) does not provide any testing protocol that uses a medium suitable for the growth of *P. salmonis*. An advantage of the use of these statistical methods (NRI and ECOFFinder) is that the standard deviations of the distribution of MIC values they calculate for fully susceptible, wild type (WT) isolates provide a quantitative estimate of the precision of sets of laboratory generated MIC data. Smith et al. (2012) suggested that there are a number of reasons why low precision may be encountered. The use of an inadequate test protocol or imprecision in the performance of the protocol would lead to imprecision as would an excessive taxonomic diversity in the isolates examined. Low precision will also be recorded if there is heterogeneity in the susceptibilities of the isolates that have been assumed to be WT. This is liable to occur if the distribution of MIC values for fully susceptible isolates overlaps with the distribution of isolates manifesting a slight reduction in susceptibility. Smith et al. (2012) have suggested that for setting acceptable epidemiological cut-off values a lower limit for the precision of the data should be set. They suggested that such a precision limit could be set following a consideration of the standard deviation for presumptive WT distributions previously calculated for a sufficient number of species/antibiotic data sets.

This study provides a novel and validated protocol for performing MIC tests to determine the susceptibility of *P. salmonis* strains to oxytetracycline and florfenicol and investigates the precision of the data generated. It also compares the performance of NRI and ECOFFinder as tools for calculating epidemiological cut-off values for interpreting the meaning of the data obtained.

## Materials and Methods

### Bacterial Isolates

A total of 58 isolates of *P. salmonis* were recovered from Piscirickettsiosis outbreaks occurred in various salmon farms as well as different periods of time and geographical locations along the South of Chile. Sampling of the fish was done according to a standard protocol (OIE, 2016). Isolates were recovered from internal organs, including brain, kidney, or liver of farmed salmonid species, Atlantic salmon (*Salmo salar*), Pacific salmon (*Oncorhynchus kisutch*), or rainbow trout (*Oncorhynchus mykiss*). The identity of the isolates was confirmed by amplifying their 16S rRNA gene sequences according to the method described by Karatas et al. (2008).

The strains, *Escherichia coli* ATCC 25922, *Aeromonas salmonicida* subsp. *salmonicida* ATCC 33658 and *P. salmonis* ATCC VR-1361 were included as quality control reference strains. The *E. coli* and *A. salmonicida* strains are those used in CLSI standardized susceptibility test protocols (CLSI, 2014a). The *P. salmonis* reference strain was isolated from the kidney of Coho salmon (*O. kisutch*) in 1989 (Fryer et al., 1990). As this date preceded the extensive use of antibiotics to control this species it was thought reasonable to assume that it could serve as a representative of the fully susceptible members of this species.

### Medium and Culture Conditions

All isolates were isolated and cultured at 18°C for up to 8 days on a solid medium specially formulated by the authors for the recovery and growth of *P. salmonis*. The medium was composed of trypticase soy agar (40 g L^-1^), sodium chloride (15 g L^-1^), D-glucose (10 g L^-1^), L-cysteine hydrochloride (1 g L^-1^), and supplemented with 5% defibrinated sheep blood and 5% calf bovine serum. When isolates were grown, discrete colonies were selected to be used in the broth microdilution assay to determine the MIC, using the specially formulated medium for this purpose, named IFOP-PsM11. The medium IFOP-PsM11 was prepared as follows: trypticase soy broth (25 g L^-1^) and sodium chloride (15 g L^-1^) were dissolved in 950 mL of distilled water, the solution was sterilized by autoclaving at 121°C for 15 min, and then was cooled at room temperature until 45°C and finally D-glucose (10 g L^-1^), L-cysteine hydrochloride (1 g L^-1^) and 5% calf bovine serum (Gibco Labs), were added aseptically and the volume was made up to 1 L.

### Minimum Inhibitory Concentrations

MICs of florfenicol and oxytetracycline of *P. salmonis* isolates were determined using a broth microdilution method. In general, the protocol used followed that recommended by CLSI (2014a,b). However, some modification to this protocol, notably with respect to the media and incubation conditions, were required by the growth characteristics of this species.

The solutions of the antibacterials florfenicol (Sigma-Aldrich) and oxytetracycline (Sigma-Aldrich) were prepared as follows: a stock solution of the antibiotic to be used was prepared at a concentration of 5,120 μg mL^-1^ (using solvents recommended by the CLSI guidelines), and 18 twofold dilutions of both antimicrobials were prepared in 96-well microplates obtaining final concentrations ranging from 0.0019 to 256 μg mL^-1^.

To prepare dilutions, 0.1 mL of IFOP-PsM11 broth for *P. salmonis* was deposited in each microplate well. Then, in the first column of the microplate, 0.1 mL of the stock solution was deposited (pre-diluted to 512 μg mL^-1^), as the well contained 0.1 mL of broth; the final dilution of the first column was 256 μg mL^-1^. Lastly, by using a multichannel pipette, 0.1 mL was taken from the wells of the first column and transferred to the second column, homogenized with the 0.1 mL of broth that already contained the well, and so on. Thus, each following well contained half the concentration of the prior well.

Each bacterial suspension was standardized at a cell density of 1–2 × 10^8^ colony-forming units mL^-1^ (CFU mL^-1^), by using a 0.5 McFarland standard, and each well was inoculated with 10 μL of the bacterial suspension. Two columns were left with the purpose to be used as a positive control (without antibiotic and inoculated with *P. salmonis*) and a negative control (without antibiotic and not inoculated with the bacterium). Each MIC assay was performed twice and the MIC value was considered as the lowest concentration of the antibiotic causing an absence of bacterial growth, after a period of 7–10 days at 18°C.

### Additional MIC Data Sets

The MIC data for 27 species/antibiotic combinations obtained in studies in a single laboratory was accessed from the EUCAST website^4^. These data were obtained using the International Standards Organisation protocol ISO 20776-1^5^.

### Data Analysis

Two analytical methods, NRI and ECOFFinder were used to calculate epidemiological cut-off values. The NRI method was performed using the MS Excel spreadsheet available on-line at http://www.bioscand.se/nri/. The NRI method was used with permission from the patent holder, Bioscand AB, Täby, Sweden (European patent No. 1,383,913, US Patent No. 7,465,559).

The ECOFFinder analysis (see text footnote 3) developed from the work of Turnidge et al. (2006), was performed by using the latest version of the MS Excel spreadsheet ECOFFinder XL 210v2.0.xism (J. Turnidge, personal communication).

### Terminology

In referring to internationally accepted, consensus-based epidemiological cut-off values, CLSI use the abbreviation ECV, whereas EUCAST use the abbreviation ECOFF. For the epidemiological cut-off values established in this work, which have yet to be considered by any national or international agency, the abbreviation CO_WT_ will be used.

In referring to the categories generated by these cut-off values we followed the terminology recommended by Silley (2012). He argued that the terms sensitive and resistant should not be used to refer to the categories generated by the application of epidemiological cut-off values. Rather isolates manifesting a MIC below the cut-off value and, therefore, indistinguishable from fully susceptible members of their species should be termed WT. Those manifesting MIC values above the cut-off should be referred to as non-wild type (NWT).

## Results

### Quality Control

A protocol suitable for the testing of *P. salmonis* has not yet been developed by CLSI, and therefore, no acceptable ranges for reference control strains have been set for any protocol suitable for the susceptibility testing of *P. salmonis*. The MIC data generated for *E. coli* ATCC 25922 and *A. salmonicida* ATCC 33658 using the test protocol developed in this work could not, therefore, be compared to any strictly relevant acceptable ranges. The 16 MIC values (4 per strain/antibiotic combination) determined for these strains were, however, all within the acceptable ranges published for other protocols in the CLSI supplement VET03-VET04-A2 (CLSI, 2014b). The range of florfenicol MICs for the *P. salmonis* ATCC VR-1361 control strain was 0.0625–0.25 μg mL^-1^ and for OTC they were between 0.0312 and 0.125 μg mL^-1^.

### EUCAST Data Sets

A total of 27 single laboratory MIC data sets published by EUCAST were analyzed by both the NRI and ECOFFinder analytical methods. From the NRI analyses the standard deviations of the log_2_ normalized WT distributions for these data sets were calculated. The 95th percentile of these values was 1.092 log_2_ μg mL^-1^. From the ECOFFinder analysis the log_2_ standard deviations of the 27 best-fit lines was calculated. The 95th percentile of these values was 0.979 log_2_ μg mL^-1^. Valid and accurate CO_WT_ values cannot be set from excessively imprecise MIC data. For the purpose of setting CO_WT_ these 95th percentile values were taken as the suggested lower limits of acceptable precision.

### *Piscirickettsia salmonis* Florfenicol Data

**FIGURE 1:**
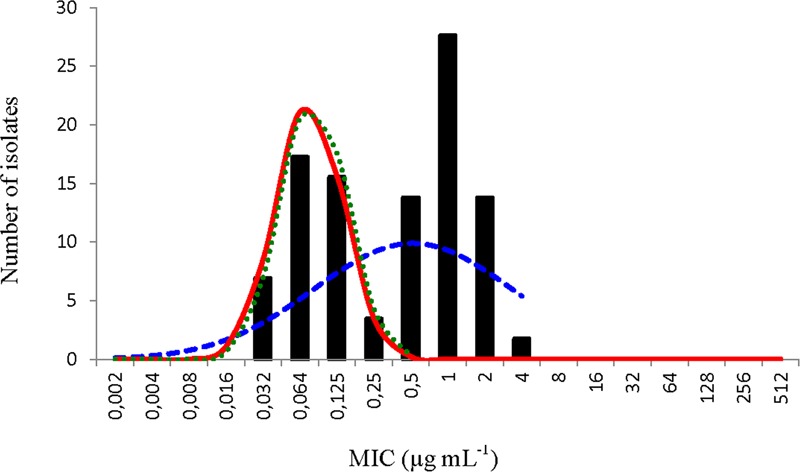
Distribution of minimum inhibitory concentration (MIC) values of florfenicol of 58 *Piscirickettsia salmonis* isolates. The continuous red line represents the distribution of the normalized MIC values for the putative wild type (WT) observations calculated by the normalized resistance interpretation (NRI) analysis. The dashed blue and dotted green lines represent the best fit line of the distribution of putative WT observations calculated by the ECOFFinder and modified ECOFFinder analyses, respectively.

The NRI analysis treated the distribution of MIC values determined for FFN as bimodal. The distribution of the normalized values for the putative WT observation calculated by NRI analysis is shown in **Figure 1**. The standard deviation of this distribution was 0.896 log_2_ μg mL^-1^, well below the precision limit (1.092 log_2_ μg mL^-1^). This analysis calculated a CO_WT_ of ≤0.25 μg mL^-1^ and categorized 33 (56%) of the isolates as NWT.

The application of ECOFFinder to these data (**Figure 1**) also calculated a CO_WT_ of ≤0.25 μg mL^-1^ and the standard deviation (0.848 log_2_ μg mL^-1^) was also well within the suggested precision limit (0.979 log_2_ μg mL^-1^). The results obtained for the control strain *P. salmonis* ATCC VR-1361 which, because of the early date of its isolation would be expected to be fully susceptible to florfenicol, were all below the cut-off value of ≤0.25 μg mL^-1^.

The most plausible interpretation of the florfenicol MIC data generated in this work was that it was composed of observations from two overlapping sub-populations. One sub-population, with a modal MIC of 0.0625 μg mL^-1^, which was fully susceptible and other, with a modal value of 1 μg mL^-1^, that manifested reduced susceptibility.

### *Piscirickettsia salmonis* Oxytetracycline Data

**FIGURE 2:**
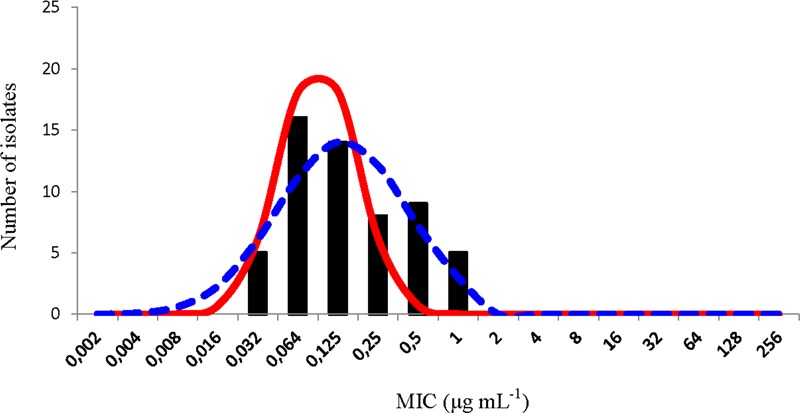
Distribution of minimum inhibitory concentration (MIC) values of oxytetracycline of 58 *Piscirickettsia salmonis* isolates. The continuous red line represents the distribution of the normalized MIC values for the putative wild type (WT) observations calculated by the normalized resistance interpretation (NRI) analysis. The dashed blue represents the best fit line of the distribution of putative WT observations calculated by the ECOFFinder analysis.

The NRI analysis treated the distribution of these oxytetracycline MIC values as bimodal. The distribution of the normalized values for the putative WT observation calculated is shown in **Figure 2**. The standard deviation of this distribution, 0.951 log_2_ μg mL^-1^, was within the suggested limit. This analysis gave a CO_WT_ of ≤0.5 μg mL^-1^ and categorized five isolates (9%) as NWT.

The ECOFFinder analysis treated the distribution of these oxytetracycline MIC values as unimodal. The best-fit line of the distribution of putative WT observations calculated by this analysis is shown in **Figure 2**. The CO_WT_ value calculated was ≤1 μg mL^-1^ and the application of this value did not categorize any isolate as NWT. However, the log_2_ standard deviation of the WT distribution calculated by this method was 1.335 log_2_ μg mL^-1^, that is well in excess of the suggested precision limit. Thus, CO_WT_ of 1 μg mL^-1^ cannot be considered valid and the treatment of the data as unimodal has to be treated as probably erroneous.

The most plausible interpretation of the OTC data is that it was also composed of observations from two overlapping sub-populations. One sub-population, with a modal MIC of 0.0625 μg mL^-1^, which was fully susceptible and other, with a modal value of 0.5 μg mL^-1^, that manifested reduced susceptibility.

## Discussion

### Antibiotic Use in Chilean Salmon Farms

The amount of antibiotics being used in Chilean salmon farming is a cause for serious concern and, as a matter of urgency steps must be taken to reduce this use. Smith (2012) has argued that improved husbandry, the availability and use of effective vaccines or alternative therapeutic agents can contribute to such a reduction. He also argued that the use of antibiotics would be reduced if each administration of an antibiotic should be associated with a determination of the susceptibility of the target bacterium. These susceptibility tests can be performed and correctly interpreted only when standardized test susceptibility protocols and interpretive criteria for the data they generate are available. The adoption of standard test protocols and the protocol-specific cut-off values represent, therefore, an essential first step in the movement toward more rational and prudent use of antibiotics to control Piscirickettsiosis.

### Developing a Standard Susceptibility Test Protocol

The performance of the protocol developed in this work clearly demonstrated that it was capable of generating MIC data of adequate precision from all the isolates tested. Therefore, it has the performance characteristics required to be considered a standard protocol. However, two other protocols for the susceptibility testing of *P. salmonis* have been suggested. Yáñez et al. (2014) tested only three isolates making any comparison with their data of limited value. Henríquez et al. (2015) performed a much larger study and it would seem worthwhile to compare the information available on the performance characteristics of the protocols they used and the protocols used in this work.

Different protocols would be expected to generate different MIC values and, therefore, different cut-off values. Such differences would be of little significance in comparing their relative performance. The primary aim of susceptibility testing is to categorize isolates on the basis of the MIC values they manifest as either WT or NWT. The degree of separation between the distribution of MICs for WT isolates and for NWT isolates would, therefore, represent a significant basis for a comparison of the performance of different test protocols. This study and that of Henríquez et al. (2015) studied the susceptibility of Chilean isolates of *P. salmonis*, it would seem reasonable to examine the relative degree of WT/NWT separation they achieved.

With respect to florfenicol both studies characterized their data as representing an overlapped bimodal distribution. The degree of separation of the categories obtained in this work was, however, significantly greater than that reported by Henríquez et al. (2015). In this work, the modal values of the WT (0.0625 μg mL^-1^) and NWT (1 μg mL^-1^) were separated by four dilutions. In the work of Henríquez et al. (2015), the equivalent modal values were 0.25 and 1 μg mL^-1^ and the separation was only two dilutions.

Analysis of the degree of separation of WT and NWT with respect to oxytetracycline is more complex. The most plausible interpretation of the oxytetracycline MIC data generated in this work was that it represented an overlapping bimodal distribution with the modal value of the more susceptible group being 0.0625 μg mL^-1^ and that of the less susceptible 0.5 μg mL^-1^. In contrast, a visual examination of the oxytetracycline data published by Henríquez et al. (2015) showed an essentially unimodal distribution with 98% of the isolates manifesting MIC values within the range of 0.125–1 μg mL^-1^. However, when these data were analyzed using ECOFFinder (see text footnote 3) and NRI (see text footnote 2), the standard deviation of the WT distributions (1.15 and 1.05 log_2_ μg mL^-1^, respectively) were above the suggested precision limits (0.95 μg mL^-1^ and 1.01 log_2_ μg mL^-1^, respectively). One possible explanation of these high standard deviations is that the single modal group that Henríquez et al. (2015) identified in their observed MIC distribution was obtained, in fact, from two sub-populations that were heterogeneous with respect to their susceptibility to oxytetracycline and whose MIC distribution showed extensive overlap. If this explanation is correct it suggests that the protocol used by Henríquez et al. (2015) lacked the sensitivity to generate data that separated these two sub-populations.

Thus, on the basis of these comparisons, the protocol developed in this work manifested a greater separation of the MIC values determined for putative WT and NWT isolates than that used by Henríquez et al. (2015).

### Applications of a Standardized Susceptibility Protocol

The Aquatic Animal Health Code of the World Animal Health Organisation^6^ recommends that the relevant authorities should initiate programs to monitor antibiotic susceptibility in the aquatic animal pathogens that are of importance in their area. With respect to Chile, *P. salmonis* represents the most significant aquatic animal pathogen. Therefore, in this country, the adoption of the standardized consensus testing protocol and its associated epidemiological cut-off values required by a program to monitor *P. salmonis* susceptibility should be given priority.

The evidence presented in this paper demonstrates that the protocol developed in this work has the properties that would be required of a standard protocol. It, therefore, deserves serious consideration as the consensus standard susceptibility testing protocol for *P. salmonis*. The WT and NWT classification of the bacterial populations generated applying by the cut-off values to data generated by this protocol would represent an important tool in the surveillance of the trends in the antimicrobial^6^ susceptibility of *P. salmonis* in the salmon production. These data would allow improved management of the application of antimicrobial treatments in the production of salmonids aimed at prolonging the therapeutic efficacy of agents and avoiding therapy failures.

### Significance of *P. salmonis* Isolates Demonstrating Small Reductions in Susceptibility

In this work, a high frequency of *P. salmonis* strains not fully susceptible to florfenicol (56%) contrasted to a lower frequency strains not fully susceptible to oxytetracycline (9%). This reflects observation that florfenicol is predominately the drug of first choice to treat this pathogen in Chilean salmon farms. However, given the amount of antibiotics that have been used in attempts to control *P. salmonis* infections, it is somewhat surprising that neither this study nor that of Henríquez et al. (2015) detected, with any significant frequency, strains of *P. salmonis* manifesting large reductions in susceptibility to these agents. Rather, data obtained in both this study and that of Henríquez et al. (2015) suggests that the response of this species to the considerable selective pressure has experienced the emergence of isolates with small reductions in susceptibility. The clinical significance of these small reductions in susceptibility is, therefore, an important issue that must be addressed future studies.

## Author Contributions

SC-L participated in the conception and design of the work, contributed to organize the different research activities and writing and editing the manuscript. PS contributed to the analyzing of the MIC data and to the writing and editing of the manuscript. PO supervised the laboratory work with the bacterial isolates and the MIC tests. ML contributed to the performance of the isolates cultivation and making the MIC analysis. WF contributed to the development of the software used to perform NRI analyses. CM contributed with the microbiological advise and to the editing of the manuscript. All authors read and approved the final manuscript.

## Conflict of Interest Statement

The authors declare that the research was conducted in the absence of any commercial or financial relationships that could be construed as a potential conflict of interest.

## Footnotes

1 www.oie.int/international-standard-setting/aquatic-code/

2 http://www.bioscand.se/nri/

3 clsi.org/standards/micro/ecoffinder/

4 http://www.eucast.org/mic_distributions_and_ecoffs/

5 http://www.iso.org/iso/iso_catalogue/catalogue_tc/catalogue_detail.htm?csnumber=41630

6 http://www.oie.int/international-standard-setting/aquatic-code/access-online
